# When a Headache Means More: A Case Report of Acute Spontaneous Subdural Hematoma After Spinal Anesthesia for Caesarean Section

**DOI:** 10.7759/cureus.37917

**Published:** 2023-04-21

**Authors:** Abhijit R Chandankhede, Snehal D Thombre

**Affiliations:** 1 Neurosurgery, Shree Siddheshwar Multispeciality Hospital, Dhule, IND; 2 Anesthesiology, Shree Siddheshwar Multispeciality Hospital, Dhule, IND

**Keywords:** complication of neuroaxial anesthesia, burr hole drainage, chronic subdural hematoma (csdh), anesthesia spinal, acute subdural hematoma

## Abstract

This case report describes a 30-year-old woman who developed an acute spontaneous subdural hematoma (SDH) after receiving intraspinal anesthesia for a cesarean section, presenting with only headache as an initial symptom. The purpose of the report is to emphasize the importance of considering acute spontaneous SDH as a potential complication of intraspinal anesthesia in patients presenting with headache, even in the absence of other neurological deficits, and the need for prompt recognition and management of this condition, as early intervention can significantly improve outcomes. The report also highlights the importance of informed consent and patient education about the potential risks and benefits of different types of anesthesia during cesarean section. The discussion includes the pathophysiology of subdural hematoma after spinal anesthesia, potential causes of severe headache, and the importance of distinguishing between neurological symptoms of intracranial hypotension, post-dural puncture headache (PDPH), and subdural hematoma. The patient underwent burr hole evacuation after the subdural hematoma converted completely to chronic, with no neurological abnormality or recurrence till now.

## Introduction

Acute spontaneous subdural hematoma (SDH) is a rare but potentially life-threatening complication of intraspinal anesthesia that can present with a variety of symptoms. In some cases, patients may initially present with only a headache, making early diagnosis and intervention challenging [1]. In this case report, we present a case of a 30-year-old woman who developed acute spontaneous SDH after receiving intraspinal anesthesia for a cesarean section and presented with only a headache as an initial symptom.

This case report highlights the importance of considering acute spontaneous SDH as a potential complication of intraspinal anesthesia in patients presenting with headaches, even in the absence of other neurological deficits. It underscores the need for prompt recognition and management of this condition, as early intervention can significantly improve outcomes.

## Case presentation

A 30-year-old lady without any co-morbidity who had undergone a cesarean section eight days back presented to us with a severe headache. She had received spinal anesthesia for the procedure. Her pre-anesthetic checkup was normal. The spinal needle used for the spinal anesthesia at level L3-L4 space was of size 28G, the spinal puncture was done in a single attempt, and the cerebrospinal fluid (CSF) flow was normal without any blood or blood-tinged CSF. The procedure was uneventful, without any immediate complications. She delivered a healthy female child. On the second postoperative day, she started complaining of a headache that was not localized and was constant. The headache did not subside after the pain medication, like non-steroidal analgesics, and worsened over the next 12 hours. She started having repeated vomiting after 24 hours. And she was put on antiemetics and analgesics with intravenous fluids considering this headache to be a postspinal dural headache. Over the next few days, the intensity of the headache fluctuated, but she was never free of headaches for eight days after the procedure. She was discharged and was prescribed analgesics. She reported to our outpatient department on the eighth postoperative day with the complaint of a worsening headache.

**Figure 1:**
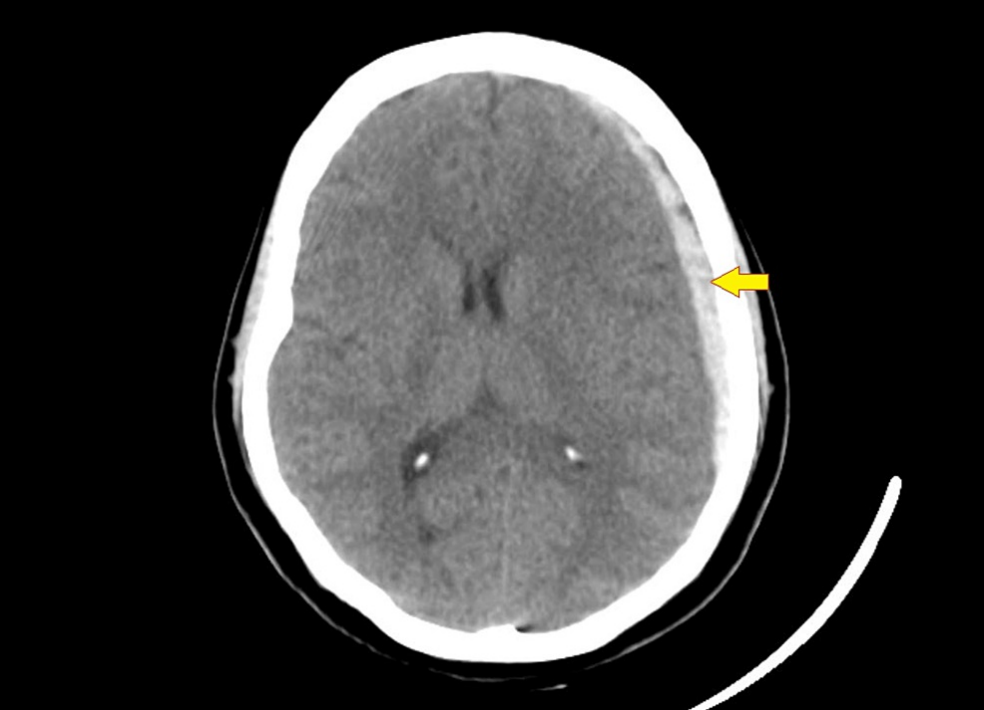
Left frontotemporoparietal subacute SDH with moderate mass effect Arrow shows the location of SDH SDH - subdural hematoma

On examination, she had a Glasgow Coma Score (GCS) 15; there were no significant neurological signs suggestive of raised intracranial pressure or any signs of mass effect. All cranial nerves were normal on examination, and her motor and sensory examination did not reveal any signs of abnormality. She underwent a computed tomogram of the brain to screen for any abnormality. She had a large left subacute subdural hematoma with moderate mass effect with a midline shift of 5 mm (Figure 1).

She was admitted to intensive care and started on intravenous mannitol and antiepileptics. She was monitored carefully for the next five days for any change in the neurological status and with repeated CT brain to evaluate the change in the size of the subdural hematoma and pressure effect. The hematoma did not increase in size or the mass effect. The neurological status of the patient also remained intact during that course.

On the fifth day of admission to the intensive care unit, the CT brain showed signs of subdural hematoma converting to chronic subdural hematoma with decreased density. The patient was again followed up for the next six days without any change in the neurological status. However, during this period, the hematoma changed from subacute to chronic and decreased in density over time.

The decision was taken to wait for the hematoma to completely convert to chronic and then drain it by small burr hole craniotomy if the neurological condition remains intact.

For the next four days, the hematoma converted completely to chronic with hypodense right frontotemporoparietal collection with the same midline shift of 5 mm to the left.

**Figure 2:**
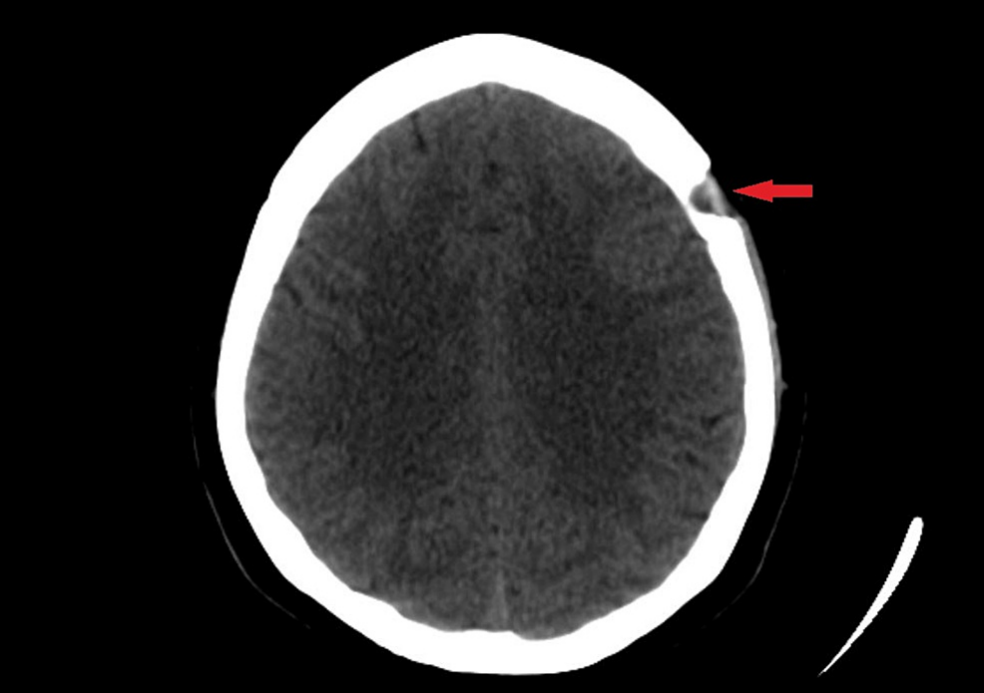
Postoperative CT brain showing complete evacuation of left SDH Arrow shows the location of the left frontal burr hole. SDH - subdural hematoma

On day 23 after the cesarean section, the decision was taken to drain the chronic subdural hematoma with a left frontal burr hole. The burr hole of size 19mm was created on the frontal bone to expose the dura. The dura was coagulated and opened in a cruciate manner to drain the chronic subdural hematoma. Subdural irrigation was done with a silicone tube to completely evacuate the hematoma (Figure 2). A subdural drain was kept for the next 24 hours to drain any residual collection. The procedure and the postoperative period were uneventful.

The patient was discharged on the third postoperative day. The follow-up brain scan showed complete evacuation of the hematoma without any residual collection or recurrence. The patient resumed her activity normally from postoperative day five. Currently, the patient is being followed regularly and has shown no signs of any neurological abnormality or recurrence of the hematoma so far.

## Discussion

Subdural hematoma is an unusual complication of dural puncture for spinal anesthesia. It is commonly seen with large bore needles used for spinal anesthesia, patients with coagulopathy, and the preoperative use of anticoagulants. The acute cranial subdural after spinal anesthesia for the caesarian section is also less commonly reported in the literature [2,3]. The pathophysiology of subdural hematoma after spinal anesthesia is much debated. Accepted possibilities are the traumatic tap leading to a leak of blood in the cerebrospinal fluid (CSF) space leading to subdural hematoma. Much accepted mechanism is intracranial cerebrospinal fluid hypovolaemia and low intracranial pressure causing stretching and tearing of bridging veins between the cortex and dural venous sinuses [4,5]. A pregnant patient who experiences a severe headache after spinal anesthesia may have several potential causes, such as a post-dural puncture headache or intracranial pathologies. It is crucial to maintain a high level of suspicion and promptly investigate patients who have persistent headaches to save their lives.

Distinguishing between the neurological symptoms of intracranial hypotension, post-dural puncture headache (PDPH), and subdural hematoma (SDH) is a challenging task. In Amorim et al.'s study on SDH cases, 89% of patients who reported headaches also experienced accompanying symptoms such as vomiting, diplopia, cognitive changes, altered mental status, or focal neurological signs [6]. The International Society of Headaches has given a list of criteria to differentiate PDPH from other serious causes of headaches like subdural hematoma. According to these criteria, in PDPH, the pain worsens or develops within 15 minutes of assuming an upright position and improves within a similar period after the individual lies down, and it develops within five days after the puncture and disappears spontaneously within one week, or up to 48 hours after an epidural blood patch is employed. Any deviation from such a pattern of headache should arouse a suspicion of a serious cause of headache [7].

In such suspicious cases, it is prudent to get a non-contrast CT brain done to confirm the presence of any intracranial pathology. When it comes to detecting subdural hematoma (SDH), head CT scans can easily visualize acute cases as high-density crescent-shaped collections across the hemispheric convexity. Meanwhile, subacute and chronic SDH can appear as isodense and hypodense crescent-shaped lesions, respectively. Although head CT scans can detect SDH, brain MRI is more sensitive in detecting intracranial hemorrhage, including small SDH and tentorial and interhemispheric SDH. MRI has an added advantage in detecting these conditions compared to head CT scans [8].

The treatment of acute subdural hematoma requires urgent evacuation of the hematoma through craniotomy in the event of progressive neurological deterioration. However, the patient experienced only headaches and no other neurological symptoms in the present case. Therefore, a decision was made to wait for the hematoma to become a chronic subdural hematoma to avoid the complications associated with craniotomy. This case report highlights the significance of vigilant observation and frequent neurological assessments in similar cases and emphasizes the potential for less invasive surgical options to benefit the patient.

## Conclusions

In conclusion, this case report highlights the importance of considering acute spontaneous subdural hematoma (SDH) as a potential complication of spinal anesthesia in patients presenting with headaches, even in the absence of other neurological deficits. The report also emphasizes the need for prompt recognition and management of this condition, as early intervention can significantly improve outcomes. The pathophysiology of subdural hematoma after spinal anesthesia is much debated, and distinguishing between the neurological symptoms of intracranial hypotension, post-dural puncture headache (PDPH), and subdural hematoma (SDH) is crucial to saving the patient's life. This case report also highlights the possibility of providing a less morbid surgical approach if the patient is neurologically stable. Further studies are warranted to better understand the incidence, risk factors, and optimal management of acute spontaneous subdural hematoma after intraspinal anesthesia.
